# Influence of bark on fuel ethanol production from steam-pretreated spruce

**DOI:** 10.1186/s13068-015-0199-x

**Published:** 2015-02-08

**Authors:** Balázs Frankó, Mats Galbe, Ola Wallberg

**Affiliations:** Department of Chemical Engineering, Lund University, PO Box 124, Getingevägen 60, Lund, SE-221 00 Sweden

**Keywords:** Ethanol, Softwood, Spruce, Bark, Steam pretreatment, SSF, SHF

## Abstract

**Background:**

Bark and bark-containing forest residues have the potential for utilization as raw material for lignocellulosic ethanol production due to their abundance and low cost. However, the different physical properties and chemical composition of bark compared to the conventionally used wood chips may influence the spruce-to-ethanol bioconversion process. This study assesses the impact of bark on the overall bioconversion in two process configurations, separate hydrolysis and fermentation (SHF) and simultaneous saccharification and fermentation (SSF), utilizing steam-pretreated spruce bark and wood mixtures.

**Results:**

Mixtures of different proportions of spruce bark and wood chips were subjected to SO_2_-catalyzed steam pretreatment at 210°C for five minutes, which has been shown to be effective for the pretreatment of spruce wood chips. The final ethanol concentration was the highest without bark and decreased significantly with increasing proportions of bark in both process configurations. However, this decrease cannot be attributed solely to the lower availability of the carbohydrates in mixtures containing bark, as the ethanol yield also decreased, from 85 to 59% in SSF and from 84 to 51% in SHF, as the mass fraction of bark was increased from 0 to 100%.

**Conclusions:**

The results show that it was significantly more difficult to hydrolyse spruce bark to monomeric sugars than wood chips. Bark had an adverse effect on the whole bioconversion process due to its lower enzymatic hydrolyzability. On the other hand, bark inclusion had no detrimental effect on the fermentability of steam-pretreated spruce wood and bark mixtures. It was also observed that lower amounts of inhibitory degradation products were formed during the steam pretreatment of spruce bark than during the steam pretreatment of wood chips.

## Background

The driving force behind the exploitation of renewable energy sources is the necessity to shift from a fossil-fuel dependent economy to one based on renewable resources. Biomass can be used to efficiently produce renewable liquid or gaseous fuels, providing alternatives to fossil fuels [1]. Ethanol, for instance, is already being produced from sugar and starch crops, and is used worldwide, as a consequence of policies promoting ethanol production [2-5]. However, the controversy of ethanol production from sugar and starch crops (first-generation ethanol) has led to the development of technologies employing lignocellulosic biomass as raw material [6-8].

The utilization of lignocellulosic biomass to produce ethanol provides an alternative to sugar and starch crops. However, the additional cost of the lignocellulosic ethanol production process, resulting from the necessity of pretreatment and enzymes for lignocellulosic biomass refining, has led to limited profitability in comparison with sugar- and starch-based ethanol production [9]. Thus, there is a need to further decrease the production cost of lignocellulosic ethanol in order for it to become competitive with the first-generation ethanol [10].

One possible means of cost reduction is to utilize abundant low-cost lignocellulosic raw materials such as bark [11]. Bark and bark-containing forest residues could serve as a potential feedstock for ethanol production, although bark is considered to be an inferior raw material to higher-value wood chips due to its composition. Compared with spruce wood chips, spruce bark has a lower content of carbohydrates, and contains significantly more extractives and ash [12]. The lower content of carbohydrates results in decreased sugar concentration after hydrolysis, and thus a lower ethanol concentration after fermentation. The pre-hydrolyzates obtained from pretreated bark can contain elevated amounts of water-soluble extractives and polyphenols, which may have inhibitory effects on fermenting microorganisms and cellulolytic enzymes [13-15]. Therefore, the combined utilization of bark and wood chips for ethanol production might pose an even greater challenge than the use of softwood chips, which is already demanding. In this case, not only the inherent recalcitrance of the material must be overcome, but also the problems resulting from the significant difference in the physical properties and chemical composition of bark and wood chips. However, if these limitations could be overcome, then abundantly available, low-value forestry residues could be exploited, and existing spruce-to-ethanol production processes could be simplified by not having to debark the material. Ultimately, it is likely that forestry residues available for bioethanol production will contain varying amounts of bark, and it is therefore important to investigate the effects of bark on production processes previously optimized for wood chips only.

Previous studies have mainly focused on the effects of bark on fermentability. Boussaid *et al*. found that including bark led to decreased fermentability of pre-hydrolyzates prepared under low-severity pretreatment conditions, while pre-hydrolyzates prepared under higher severity conditions could be fermented comparably well to ethanol when 9% bark was included [13]. Similar results were obtained by Robinson *et al*., who found that up to 30% bark, on a dry basis, had negligible effects on the fermentability of pre-hydrolyzates obtained from SO_2_-catalyzed steam-exploded Douglas fir whitewood [16]. Although the enzymatic hydrolyzability of bark has been investigated previously [17], there are few reports on the influence of bark on enzymatic hydrolysis and the overall ethanol yield of the ethanol bioconversion process when performed at higher water-insoluble solids (WIS) content [18]. Enzymatic hydrolysis and fermentation must be performed at a higher WIS loading in order to increase the ethanol concentration after fermentation, which is essential to reduce the cost of distillation and thus the marginal production cost production [19].

The aim of the present study was to assess the feasibility of utilizing bark together with spruce wood chips for the fermentative conversion of biomass to ethanol at 10% WIS content, using a commercial enzyme cocktail to hydrolyse the steam-pretreated material, and an industrial strain of *Saccharomyces cerevisiae* as the fermenting microorganism. Spruce bark mixed with wood chips at different ratios ranging from 0 to 100% were subjected to SO_2_-catalyzed steam pretreatment at 210°C for five minutes, which has previously been shown to be effective for spruce wood chips [20]. The effects of bark inclusion on the spruce-to-ethanol bioconversion process were investigated by performing separate hydrolysis and fermentation (SHF) and simultaneous saccharification and fermentation (SSF) of the steam-pretreated wood and bark mixtures.

## Results and discussion

### Steam pretreatment of wood and bark mixtures

The composition of the spruce wood chips and bark is given in Table 1. The content of carbohydrates, with the exception of arabinan, was lower in the bark than in the wood chips. The amount of hexose sugars available in the bark feedstock was only about half of that in the wood feedstock. Even though only neutral carbohydrates were analyzed, spruce bark also contains a significant amount of other polysaccharides, such as pectin [21,22]. The bark contained significantly more extractives and ash than were found in the wood chips. The content of acid-soluble lignin may be slightly overestimated for both feedstocks due to possible interference from other non-lignin components [23].

**Table 1:** **Composition of the spruce wood and bark feedstocks as a percentage of dry matter (% of DM)**

**Feedstock**	**Carbohydrates**						**Lignin**		**Extractives** ^**c**^	**Ash**
	**Glucan**	**Xylan**	**Galactan**	**Arabinan**	**Mannan**	**Sum of neutral carbohydrates**	**ASL** ^**a**^	**AIL** ^**b**^		
Wood	42.4	5.6	1.3	0.7	9.9	59.9	7.6	26.2	3.3	0.2
Bark	23.1	3.6	0.8	4.3	3.4	35.2	13.3	20.5	28.2	2.2

^a^Acid-soluble lignin.

^b^Acid-insoluble lignin.

^c^Water and ethanol extractives.

The compositions of the water-insoluble solid fractions of the steam-pretreated materials were determined, and the results are presented in Table 2. As a result of the lower glucan content of the bark feedstock, the glucan content of the steam-pretreated mixtures decreased with increasing proportions of bark (Table 2). Detectable amounts of sugars originating from the hemicellulose were observed in the solid fraction after pretreatment of 100% bark. Steam pretreatment dissolved most of the hemicelluloses in all other steam-pretreated materials. This could be due to the higher recalcitrance of bark or the possible neutralization of the SO_2_ added to the raw material by the higher ash and extractives content of the bark feedstock. This indicates that bark requires more SO_2_ or higher severity steam pretreatment conditions to dissolve hemicellulose to the same extent as in wood chips. Interestingly, the acid-insoluble lignin content of the water-insoluble fractions increased as the bark content increased in the feedstock (Table 2), although the acid-insoluble lignin content was found to be higher in the wood chips than in the bark (Table 1). This has been found in previous studies reporting that bark contains water-soluble phenolic compounds that can condense with lignin during acid-catalyzed steam pretreatment and appear as acid-insoluble lignin in the subsequent compositional analysis [18,24]. This phenomenon could play a significant part in the structural changes of the bark during the acid-catalyzed steam pretreatment carried out at the optimal condition for the wood chips.

**Table 2:** **Composition of the water-insoluble fraction of steam-pretreated wood and bark mixtures as a percentage of dry matter (% of DM)**

**Bark content (% of DM)**	**Carbohydrates**						**Lignin**		**Ash**
	**Glucan**	**Xylan**	**Galactan**	**Arabinan**	**Mannan**	**Sum of neutral carbohydrates**	**ASL** ^**a**^	**AIL** ^**b**^	
0	54.5	n.d.	n.d.	n.d.	0.7	55.2	3.1	42.9	0.3
10	51.8	n.d.	n.d.	n.d.	0.5	52.3	2.9	43.1	0.5
30	48.4	n.d.	n.d.	n.d.	0.5	48.9	2.7	46.3	0.8
50	44.5	1.4	n.d.	n.d.	0.4	46.3	2.8	48.5	1.2
100	36.7	2.1	0.3	0.2	0.8	40.1	3.6	48.8	2.5

^a^Acid-soluble lignin.

^b^Acid-insoluble lignin.

n.d. Not detected.

**Figure 1:**
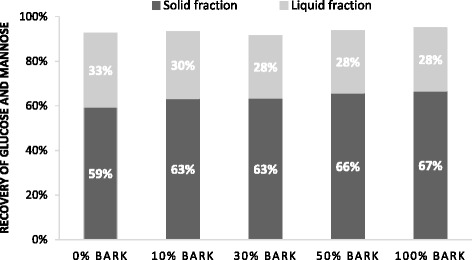
**Recovery of glucose and mannose after steam pretreatment of wood and bark mixtures.** Recovery expressed as percentage of the theoretical based on the glucan and mannan content of the raw materials.

The composition of liquid fractions obtained from the steam-pretreated materials is presented in Table 3. As a consequence of the lower carbohydrate content of bark, the concentration of total sugars (expressed in monomeric form) in the liquid fraction decreased with increasing proportions of bark, with the exception of arabinose due to the higher arabinan content of bark. As it can be seen in Figure 1, the addition of bark seemed to have a negligible effect on the overall recovery of glucose (94 to 96% for all steam-pretreated materials) and mannose (80 to 82% for all steam-pretreated materials) in the steam pretreatment step, although a lower proportion of sugars was dissolved in monomeric form in the liquid fraction as bark was added. This confirms the hypothesis that the pretreatment conditions previously found to be optimal for spruce wood chips may be too mild to overcome the recalcitrance of bark.

**Table 3:** **Composition of the liquid fraction of the steam-pretreated wood and bark mixtures**

**Bark content (% of dry matter)**	**Total sugars (expressed as monomeric sugar) (g/L)**					**Inhibitors (g/L)**				
	**Glucose**	**Xylose**	**Galactose**	**Arabinose**	**Mannose**	**HMF** ^**a**^	**Furfural**	**Formic acid**	**Acetic acid**	**Levulinic acid**
0	26.5	9.7	5.1	2.7	21.7	2.0	1.3	1.5	1.9	1.0
10	21.8	9.2	4.0	3.3	20.7	1.8	1.1	1.3	1.7	0.7
30	20.1	9.2	4.2	4.8	17.8	1.4	0.8	0.7	1.4	0.4
50	18.3	8.0	4.1	5.9	13.7	1.0	0.7	0.4	1.2	0.3
100	18.2	6.0	4.3	10.0	5.6	0.4	0.4	0.4	0.6	0.2

^a^5-Hydroxymethylfurfural.

**Figure 2:**
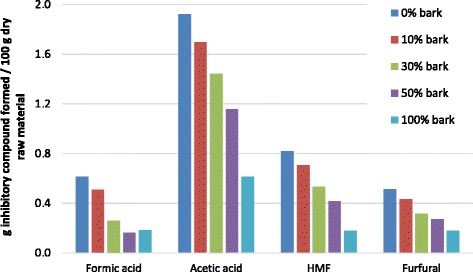
**The amounts of inhibitory compounds formed during steam pretreatment of wood and bark mixtures.** HMF, 5-hydroxymethylfurfural.

The amount of degradation products generated during steam pretreatment is a function of the severity of the pretreatment and the concentration of carbohydrates present in the feedstock. Therefore, the most likely explanations of the decreasing concentrations (Table 3), and also the amount of all measured inhibitors expressed as grams of inhibitors formed per 100 g dry raw material (Figure 2) with increasing bark inclusion, are the lower carbohydrate content and the higher recalcitrance of the bark feedstock.

### Fermentability of pre-hydrolyzates

**Figure 3:**
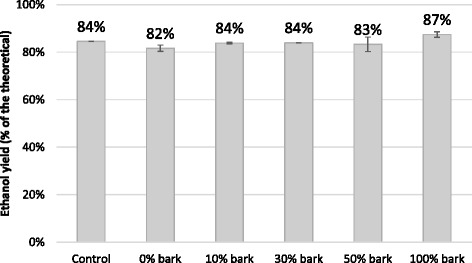
**Ethanol yields of pre-hydrolyzate fermentation.** Ethanol yields expressed as percentage of the theoretical based on glucose and mannose. Fermentation was performed with *Saccharomyces cerevisiae* at 30°C, pH 5.5 for 24 hours.

In order to evaluate the effect of the inhibitory compounds present in the liquid fractions obtained from the steam-pretreated materials, the pre-hydrolyzates were subjected to a fermentation test. As shown in Figure 3, all the pre-hydrolyzates showed similar high degrees of fermentability to ethanol, and the ethanol yields were in the same range as in the control solution, which contained only pure monomeric glucose and mannose.

Although bark contained more extractives than wood chips (Table 1), these had no detrimental effect on the fermentability of the pre-hydrolyzates. Boussaid *et al*. reported decreased fermentability of pre-hydrolyzates following low-severity steam pretreatment of bark-containing softwood, and attributed it to extractives, which were recovered in the water-soluble fraction [13]. However, additional acid hydrolysis of the pre-hydrolyzates increased the ethanol yield significantly, and they believed this to be due to the condensation and polymerization of water-soluble phenolic compounds. Moreover, they also showed that pre-hydrolyzates obtained from steam pretreatment at a severity factor above three (as defined by Overend *et al*. [25]) fermented well to ethanol despite the inclusion of 9% bark. This indicates that the relatively high severity of the steam pretreatment applied in our study also made the condensation and polymerization of water-soluble phenolic compounds possible, hence possibly eliminating their inhibitory effect. These results support previous findings that lower amounts of inhibitory compounds are generally formed during steam pretreatment of softwood bark than in the case of wood chips, and as a consequence, pre-hydrolyzates of steam-pretreated spruce bark can be fermented comparably well into ethanol [12,16,18].

### Separate hydrolysis and fermentation of wood and bark mixtures

Previous studies have mostly been devoted to the investigation of the fermentability of steam-pretreated softwood bark [12,13,16,17], while little has been reported on the effect of including bark on the enzymatic hydrolyzability of steam-pretreated softwoods. Furthermore, most previous studies have been performed at lower WIS contents [17,18]. The implementation of enzymatic hydrolysis and fermentation, either separately or simultaneously, at a higher WIS content is driven by the possible energy savings in the distillation step [26]. In order to investigate the effects of bark on enzymatic hydrolyzability and fermentability of the hydrolyzed pretreated materials separately, SHF experiments were performed at 10% WIS content. The major advantage of SHF is that hydrolysis and fermentation can be carried out under their optimal conditions. However, SHF in general requires longer overall process time in comparison with SSF [27], and the end-product inhibition of enzymes by glucose and cellobiose results in a reduced rate of saccharification [28].

**Figure 4:**
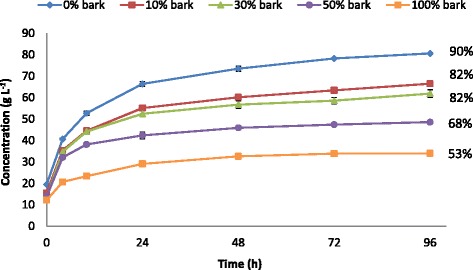
**Concentration profiles for glucose during enzymatic hydrolysis and the final glucose yields.** Enzymatic hydrolysis of steam-pretreated wood and bark mixtures was performed at 10% WIS loading, 45°C, pH 5 for 96 hours using 20 FPU/g WIS Cellic CTec3. Final glucose yields expressed as percentage of the theoretical based on all available glucose. FPU, filter paper unit; WIS, water-insoluble solids.

Figure 4 shows the concentration profiles for glucose during the enzymatic hydrolysis of steam-pretreated wood and bark mixtures and final glucose yields. The highest final glucose concentration (80.6 g/L) was achieved with no bark addition, and decreased to 34 g/L with 100% bark. Furthermore, the highest glucose yield (90% based on all available glucose) and the highest rate of hydrolysis were obtained when no bark was added. The significant extractives content of bark can be a possible explanation for the lower glucose yields with bark addition. Phenolic compounds, either in monomeric or oligomeric form, deriving from bark can have inhibitory effect on the enzymes [14,15,29], which makes the enzymatic hydrolysis of bark more challenging than in the case of spruce wood chips. As can be seen in Figure 4, the glucose yield decreased significantly with increasing proportions of bark, and reached 53% at 100% bark. However, the same trend of decreasing hydrolyzability was also observed with increasing proportions of bark at 5% WIS loading, both on whole slurry and on washed fibre (data not shown). This suggests that the underlying reason for the lower enzymatic hydrolyzability is not the inhibitory effect of phenolic compounds in the liquid phase, but rather the higher recalcitrance or the structural changes caused by the relocation of bark extractives during acid-catalyzed steam pretreatment.

Previous studies have reported higher sugar yields from bark-containing softwood or bark as the only raw material. However, these enzymatic hydrolysis experiments were performed at a lower WIS content. For instance, Kemppainen *et al*. reported between 70 and 80% yields, depending on the steam pretreatment conditions, after 48 hours of enzymatic hydrolysis of spruce bark at 1% dry matter content [18]. A glucose yield of 79.6% was obtained by Robinson *et al*. in the enzymatic hydrolysis of softwood containing bark pretreated with steam and an additional alkaline peroxide treatment [17]. Another possible way can be to use additional accessory enzymes in order to increase the yield of the enzymatic hydrolysis of bark. For instance, 24% improvement was observed in hydrolysis of spruce bark after 48 hours when pectinase enzymes were used as a supplementation to the cellulolytic enzymes [18]. Due to the presence of pectin in bark, the pectinase activity of the applied enzyme cocktail may also significantly affect the hydrolysis yields.

**Figure 5:**
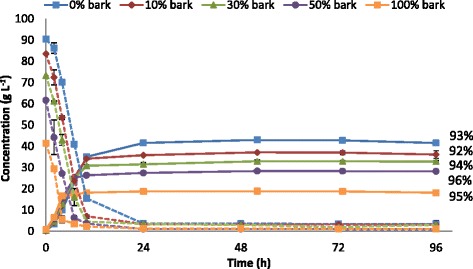
**Concentration profiles for total hexose sugars (dashed lines) and ethanol (solid lines) during fermentation and the final ethanol yields.** Fermentation of the supernatants obtained from the enzymatic hydrolysis of steam-pretreated wood and bark mixtures was performed at 10% WIS loading, 30°C, pH 5 for 96 hours using Ethanol Red yeast (5 g/L). Final ethanol yields expressed as percentage of the theoretical based on all available hexose sugars.

After the removal of the solid fraction of enzymatically hydrolyzed mixtures by filtration, the liquid fractions were subjected to fermentation. Figure 5 shows the concentration profiles for total hexose sugars and the ethanol produced during fermentation, together with the final ethanol yields (percentage of the theoretical maximum stoichiometric yield based on all available hexose sugars). The ethanol concentrations reached their maximum values after 24 hours in all cases. However, the final ethanol concentrations were considerably lower as the amount of bark was increased, due to the lower concentration of hexose sugars available for ethanol production in the enzymatically hydrolyzed bark-containing mixtures. As can be seen in Figure 5, almost all hexose sugars were consumed by the yeast and fermented into ethanol. The ethanol yields were largely unaffected by the addition of bark, and were in the same range; above 90%.

Table 4A shows the final concentrations of the substrates and products, together with the ethanol yield and the initial volumetric ethanol productivity in the SHF experiments. The volumetric ethanol productivity during the first two hours increased with bark inclusion, from 1.7 g/L⋅h to 3.2 g/L⋅h as the bark content was increased from 0 to 100%. A possible explanation for the higher initial volumetric ethanol productivity could be the lower concentration of 5-hydroxymethylfurfural (HMF) and furfural in the mixtures with higher bark content (Table 3). HMF and furfural are known to be inhibitory to yeast [30], and the fermentability of pre-hydrolyzates is significantly decreased with increasing concentrations of HMF and furfural. Taherzadeh *et al*. found that the rate of fermentation decreased considerably when the combined amount of HMF and furfural exceeded approximately 2 g/L [12]. In the present study, the HMF and furfural concentrations were highest when no bark was added to the spruce chips (1.6 g/L and 1.0 g/L, respectively), which were completely detoxified by the yeast in the first four hours of fermentation. As a consequence of the inhibitory effect of these degradation products, lower initial volumetric ethanol productivity was observed with the pretreated mixtures containing wood chips, than that for 100% bark, which contained the lowest concentrations of HMF and furfural.

**Table 4:** **Substrate, product and by-product concentrations and yields obtained from SHF (A) and SSF (B) experiments**

**A)**						
	Bark content (% of DM)	0	10	30	50	100
Enzymatic hydrolysis step in SHF	Glucose concentration (g/L)	80.6 ± 0.1	66.5 ± 0.7	61.8 ± 1.8	48.5 ± 0.5	34.0 ± 0.5
	Glucose yield (% of the theoretical)	89.8 ± 0.3	81.6 ± 1.0	81.9 ± 3.3	67.9 ± 1.1	53.3 ± 0.9
Fermentation step in SHF	Total hexose sugar concentration (g/L)	3.5 ± 0	3.1 ± 0	2.9 ± 2.1	1.0 ± 0.7	1.1 ± 0
	Glycerol concentration (g/L)	5.2 ± 0	4.5 ± 0.1	4.2 ± 0.1	3.6 ± 0	3.0 ± 0.1
	Volumetric ethanol productivity^a^ (g/L⋅h)	1.7 ± 0	2.0 ± 0	2.3 ± 1.2	2.8 ± 0.1	3.2 ± 0
	Ethanol concentration (g/L)	41.5 ± 0.8	36.1 ± 1.8	32.8 ± 0.4	28.2 ± 0.1	18.1 ± 0
	Ethanol yield (% of the theoretical)	93.1 ± 0.1	92.0 ± 0	93.8 ± 0.8	96.2 ± 0.9	95.0 ± 0.2
SHF	Overall ethanol yield (% of the theoretical)	83.6	75.1	76.8	65.3	50.7
**B)**						
	Bark content (% of DM)	0	10	30	50	100
SSF	Total hexose sugar concentration (g/L)	4.2 ± 0.3	1.9 ± 0.1	2.2 ± 0.1	2.4 ± 0.1	3.3 ± 0.1
	Glycerol concentration (g/L)	4.8 ± 0.1	4.2 ± 0.2	3.8 ± 0.1	3.4 ± 0	3.6 ± 1.4
	Volumetric ethanol productivity^a^ (g/L⋅h)	1.7 ± 0.1	1.8 ± 0.3	2.5 ± 0	2.9 ± 0.1	4.0 ± 0.1
	Ethanol concentration (g/L)	45.8 ± 0.8	39.3 ± 0.9	34.5 ± 0.4	29.4 ± 0.1	20.9 ± 0
	Overall ethanol yield (% of the theoretical)	85.4 ± 1.9	81.1 ± 2.3	77.5 ± 1.3	70.5 ± 0.4	59.3 ± 0.1

^a^Calculated for the first two hours.

SSF and SHF experiments of steam-pretreated wood and bark mixtures were performed at 10% WIS loading, pH 5 using Cellic CTec3 enzyme cocktail (20 FPU/g WIS) and an industrial *S. cerevisiae* strain, Ethanol Red (5 g/L). DM, dry matter; SHF, separate hydrolysis and fermentation; SSF, simultaneous saccharification and fermentation; WIS, water-insoluble solids.

The results of the SHF experiments showed that bark had no detrimental effects on the fermentability, which is in agreement with the results of the pre-hydrolyzates fermentation. However, the addition of bark has an adverse effect on enzymatic hydrolyzability, which limits the amount of ethanol that can be produced by the yeast in the bioconversion process.

### Simultaneous saccharification and fermentation of wood and bark mixtures

**Figure 6:**
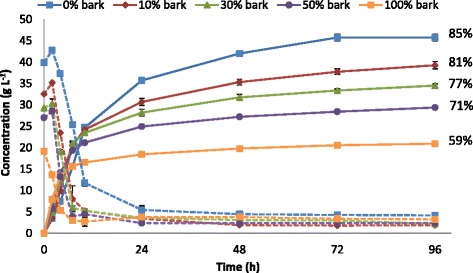
**Concentration profiles for total hexose sugars (dashed lines) and ethanol (solid lines) during SSF and the final ethanol yields.** SSF of the steam-pretreated wood and bark mixtures was performed at 10% WIS loading, 35°C, pH 5 for 96 hours using 5 g/L Ethanol Red yeast and 20 FPU/g WIS Cellic CTec3. Final ethanol yields expressed as percentage of the theoretical based on all available hexose sugars. FPU, filter paper unit; SSF, simultaneous saccharification and fermentation; WIS, water-insoluble solids.

In the SSF process configuration, enzymatic hydrolysis and fermentation are performed simultaneously in the same vessel, and the end-product inhibition during hydrolysis is minimized by the continuous conversion of glucose to ethanol by the fermenting microorganism [31]. However, enzymatic hydrolysis and fermentation are performed under sub-optimal conditions. In the present study, SSF was performed at 10% WIS content to assess the effect of including bark on both fermentability and enzymatic hydrolyzability. Figure 6 shows the concentration profiles for total hexose sugars and the ethanol produced during SSF, together with the ethanol yields obtained (percentage of the theoretical based on all available hexose sugars).

As can be seen in Figure 6, the highest final ethanol concentration (46 g/L) was obtained when no bark was added, and it decreased significantly with increasing additions of bark. However, this decrease cannot be attributed solely to the lower amount of carbohydrates available in the bark-containing mixtures, as the ethanol yield also decreased, from 85 to 59%, as the proportion of bark was increased from 0 to 100%.

As can be seen in Table 4B, the volumetric ethanol productivity during the first two hours of SSF increased from 1.7 to 4.0 g/L⋅h as the amount of bark was increased from 0 to 100%. Furthermore, accumulation of the hexose sugars was also observed during the same time period in all SSF experiments, with the exception of 100% bark, where no accumulation of hexose sugars occurred during SSF (Figure 6). Similarly to the SHF experiments, the lower concentrations of inhibitory compounds with increasing amounts of bark contributed to higher initial volumetric ethanol productivities than without bark. This indicates that the fermentability was not affected negatively by the inclusion of bark. This is also confirmed by that the addition of bark had no noticeable negative effects on the sugar utilization of the yeast in the SSF experiments (Figure 6). All the available glucose and mannose were consumed by the yeast, and only galactose was detected at low concentrations after 96 hours. Glycerol was the main by-product produced by the yeast in all cases (Table 4), and there was no significant difference in the yield of glycerol based on all available hexose sugars (approximately 4% in all SSF experiments).

The ethanol yield obtained in the SSF experiments with no bark was in the same range as reported for spruce chips in previous studies [32,33], while Kemppainen *et al*. reported an ethanol yield of 66.4% of the theoretical in SSF of sequentially hot-water extracted and steam-pretreated spruce bark [18]. This higher yield might be explained by the removal of the extractives from the bark prior steam pretreatment. Moreover, the six-hour pre-hydrolysis applied before SSF, and the possible structural differences between industrial bark and the freshly processed bark used in the present study, should not be neglected.

Comparing the two process configurations, it is apparent that SSF was superior to SHF in all cases, since SSF resulted in higher overall yields regardless of the bark content (Table 4). It is also evident that the decreased enzymatic hydrolyzability of bark is a decisive factor behind the declining ethanol yields, with increasing amounts of bark in both process configurations. Bark was found to be significantly more difficult to hydrolyse to monomeric sugars than wood chips. Lower amounts of monomeric sugars were recovered in the liquid fraction after steam pretreatment, and lower yields were observed in the enzymatic hydrolysis step with increasing bark content. Although, it appears that bark might require more severe steam pretreatment to overcome its inherent recalcitrance, the possible unfavorable structural changes in the steam-pretreated bark could also hamper the enzymatic hydrolysis. The relocation of extractives and bark lignin during the acid-catalyzed steam pretreatment might reduce the accessibility for the enzymes to cellulose, which can result in lower enzymatic hydrolyzability. Delignification methods might also be an alternative to achieve higher yields in enzymatic hydrolysis, and thus provide more sugars for ethanol fermentation; however, chemical delignification operations are expensive and would constitute an additional burden on the already sensitive economics of second-generation ethanol production [34]. Thus, further research is needed to improve the enzymatic hydrolyzability of bark in order to achieve higher yields at lower enzyme dosages.

## Conclusions

The effect of including bark in the spruce-to-ethanol production process has been assessed. The results showed that adding bark had no detrimental effects on the fermentability of steam-pretreated spruce bark and wood mixtures, and it was observed that lower amounts of degradation products were formed during the steam pretreatment of spruce bark than spruce wood chips. However, the addition of bark had an adverse effect on the whole bioconversion process due to the low hydrolyzability of bark. This was reflected by the decreasing overall ethanol yield with increasing proportions of bark in both process configurations. SSF proved to be more efficient than SHF for all wood and bark mixtures, since this process configuration resulted in higher overall yields, regardless of bark content.

## Materials and methods

### Materials

Fresh spruce, *Picea abies*, was debarked and kindly provided by a local sawmill (ATA Timber Widtskövle AB, Everöd, Sweden), together with the bark fraction. The bark and the bark-free chipped wood were further chipped using a knife mill (Retsch GmbH, Haan, Germany) and sieved in order to obtain the fraction with a size range between 2 and 10 mm. The spruce had a dry matter content of 40%, while the bark had a somewhat lower dry matter content of 35%. The raw materials were stored in plastic bags at 4°C until used.

The enzyme preparation used was Cellic CTec3, kindly provided by Novozymes A/S (Bagsværd, Denmark). The yeast used in both the SSF and SHF experiments was Ethanol Red, kindly provided by Leaf Technologies (Marcq-en-Baroeul Cedex, France). The yeast used to determine the fermentability of the pre-hydrolyzates was prepared on an agar plate from ordinary baker’s yeast, *Saccharomyces cerevisiae*, produced by Jästbolaget (Rotebo, Sweden). Vitahop, kindly provided by BetaTec (Schwabach, Germany), was used in the SSF and SHF experiments to avoid bacterial contamination. All chemicals used were of reagent grade quality.

### Feedstock preparation and steam pretreatment

The bark and wood fractions were mixed to obtain batches containing 0, 10, 30, 50 and 100% bark on a dry weight basis. Each batch had a total dry weight of 700 g. The mixtures were impregnated with gaseous SO_2_ (2.5% w/w, based on the water content of the mixtures) in tightly sealed plastic bags for 20 minutes at room temperature, and then subjected to steam pretreatment. Steam pretreatment was performed at 210°C for five minutes in a 10 L reactor (Process- & Industriteknik AB, Kristianstad, Sweden), as described previously by Palmqvist *et al*. [35]. The pretreated materials were stored at 4°C before subsequent analysis and experiments.

### Fermentation of pre-hydrolyzates

Yeast that was used to evaluate the fermentability of pre-hydrolyzates was aerobically cultivated. The inoculum culture was prepared by adding yeast cells, previously grown on a YPG agar plate (10 g/L yeast extract, 20 g/L peptone, 20 g/L glucose and 15 g/L agar) for three days at 30°C, to two 250 mL Erlenmeyer flasks, together with 70 mL of an aqueous solution containing 23.8 g/L glucose, 10.8 g/L (NH_4_)_2_SO_4_, 5.0 g/L KH_2_PO_4_ and 1.1 g/L MgSO_4_∙7 H_2_O. The solution also contained 14.4 mL/L trace element solution and 1.4 mL/L vitamin solution, prepared according to Taherzadeh *et al*. [36]. The pH was adjusted to pH 5 with 2.5 M NaOH solution. The Erlenmeyer flasks were sealed with cotton plugs and incubated at 30°C on a rotary shaker (Adolf Kühner AG, Basel, Switzerland) for 20 hours. Aerobic batch propagation was performed in a 2 L LABFORS fermentor (Infors AG, Bottmingen, Switzerland) at 30°C for 24 hours, with a working volume of 1 L. Propagation was initiated by the addition of 140 mL inoculum cultures to an autoclaved medium containing 20 g/L glucose, 22.5 g/L (NH_4_)_2_SO_4_, 10.5 g/L KH_2_PO_4_ and 2.2 g/L MgSO_4_∙7 H_2_O, 60.0 g/L trace element solution and 6.0 g/L vitamin solution. The aeration rate was 1 L/min, corresponding to 1 vvm (gas volume flow per unit working volume per minute). The stirrer speed was 700 rpm and the pH was maintained at pH 5 with 2.5 M NaOH solution. The dissolved oxygen concentration was monitored continuously with an O_2_-sensor (Mettler-Toledo GmbH, Urdorf, Switzerland). When all the sugars had been consumed as indicated by the O_2_-sensor, cultivation was stopped and the cells were harvested by centrifugation in 700 mL bottles at 3,600 × g for 10 minutes. The supernatant was discarded and the dry matter content of the harvested cells was determined. The time between cell harvesting and the initialization of the fermentation tests was less than two hours.

Fermentation tests were carried out on the pre-hydrolyzates to assess their fermentability and the extent of inhibition by the compounds formed during steam pretreatment. Pre-hydrolyzates were obtained from the steam-pretreated materials by vacuum filtration using grade five filter paper (Munktell Filter AB, Falun, Sweden). The pre-hydrolyzates were then diluted with deionized water to obtain an equivalent solids concentration (the concentration of inhibitors corresponding to an SSF with a certain WIS load) corresponding to a WIS load of 10% mass fraction. The initial concentrations of fermentable sugars were adjusted to 30 g/L glucose and 20 g/L mannose in order to obtain comparable fermentation results. A reference solution was prepared with the same sugar concentrations to serve as a control. Fermentation was performed anaerobically on a rotary shaker in shake flasks with a working volume of 100 mL, containing 0.5 g/L (NH_4_)_2_HPO_4_, 0.025 g/L MgSO_4_∙7 H_2_O and 0.2 mL/L Vitahop. Fermentation tests were conducted at 30°C and pH 5.5 for 24 hours with a yeast concentration of 5 g/L. The fermentation experiments were performed in duplicate.

### Separate hydrolysis and fermentation

Enzymatic hydrolysis of the whole pretreated slurry was performed in 2 L LABFORS bioreactors with a working weight of 1.2 kg. A WIS load of 10% mass fraction and Cellic CTec3 enzyme cocktail at a load of 20 FPU/g WIS based on the final weight, were applied. The hydrolysis experiments were performed at 45°C, with a stirring rate of 400 rpm, at pH 5 maintained with 2.5 M NaOH solution. After 96 hours of enzymatic hydrolysis the supernatants were separated by vacuum filtration using grade five filter paper (Munktell Filter AB). The supernatants obtained from the duplicates were mixed and stored at −20°C prior to fermentation.

Fermentation of the supernatant was performed in 2 L LABFORS bioreactors with a working weight of 0.55 kg. Ethanol Red yeast was added at a dry weight concentration of 5 g/L based on the final weight. The supernatant was supplemented with (NH_4_)_2_HPO_4_ solution at a concentration of 0.5 g/L and 0.125 mL/L Vitahop. Fermentation was carried out at 30°C, with a stirring rate of 250 rpm for 96 hours, at pH 5 maintained with 2.5 M NaOH solution. All experiments were performed in duplicate.

### Simultaneous saccharification and fermentation

The SSF experiments using the whole pretreated slurry were performed in sterilized 2 L LABFORS bioreactors with a working weight of 1 kg. A WIS load of 10% mass fraction, the Cellic CTec3 enzyme cocktail at a load of 20 FPU/g WIS and Ethanol Red yeast at a dry weight concentration of 5 g/L based on the final amount, were applied. The experiments were carried out at 35°C, with a stirring rate of 400 rpm for 96 hours, at pH 5 maintained with 2.5 M NaOH solution. The SSF media were supplemented with (NH_4_)_2_HPO_4_ solution at a concentration of 0.5 g/L and 0.125 mL/L Vitahop. All experiments were performed in duplicate.

### Analysis

The total solids content of biomass materials and total dissolved solids content of liquid samples were determined according to the National Renewable Energy Laboratory (NREL) standardized laboratory analytical procedure [37]. The structural carbohydrates, lignin, extractives and ash content of the solid fractions and the composition of the liquid fractions were determined according to NREL standardized laboratory analytical procedures [38-41].

All samples obtained from experiments or compositional analysis were centrifuged in 2 mL Eppendorf tubes at 16,000 × g for 10 minutes. The supernatant was filtered using 0.2 μm syringe filters (GVS Filter Technology Inc., Indiana, United States), and filtered samples were stored at −20°C prior to high-performance liquid chromatography (HPLC) analysis. Sugars, ethanol, organic acids and other by-products were analyzed using a Shimadzu LC-20 AD HPLC system equipped with a Shimadzu RID 10A refractive index detector (Shimadzu Corporation, Kyoto, Japan). Monomeric sugars were quantified with isocratic ion-exchange chromatography using an Aminex HPX-87P column with a De-Ashing Bio-Rad micro-guard column at 85°C (both from Bio-Rad Laboratories, Hercules, California, United States) using reagent grade water as the mobile phase at a flow rate of 0.5 mL/min. Ethanol, organic acids and other by-products were determined using an Aminex HPX-87H chromatography column with a Cation-H Bio-Rad micro-guard column at 50°C (Bio-Rad Laboratories, Hercules, California, United States), with a mobile phase of 5 mM sulfuric acid at a flow rate of 0.5 mL/min.

### Yield calculations

The glucose yield in the enzymatic hydrolysis experiments was calculated on the basis of total available glucose in the liquid and the solid fraction of the steam-pretreated materials. The theoretical amount of glucose released during enzymatic hydrolysis is 1.11 times the amount of glucan in the solid fraction of the steam-pretreated materials (due to the addition of water in hydrolysis). The ethanol yield is expressed as a percentage of the theoretical stoichiometric ethanol yield (0.51 g/g), based on total available hexose sugars, namely glucose, mannose and galactose, in the solid and/or the liquid fraction of the steam-pretreated materials.

## Abbreviations

AIL: Acid-insoluble lignin
ASL: Acid-soluble lignin
DM: Dry matter
FPU: Filter paper unit
HMF: Hydroxymethylfurfural
HPLC: High-performance liquid chromatography
NREL: National Renewable Energy Laboratory
SHF: Separate hydrolysis and fermentation
SSF: Simultaneous saccharification and fermentation
WIS: Water-insoluble solids
YPG: Yeast peptone glucose
